# Mechanism-Guided Spray Deposition of Rutile TiO_2_/Epoxy/ODTMS Superhydrophobic Coatings for Weather-Resistant Bamboo Sand Barriers

**DOI:** 10.3390/molecules31162773

**Published:** 2026-08-10

**Authors:** Jun Tong, Yulin Shen, Minhua Huang, Huiwen Pang, Qian Yan, Lihong Yao

**Affiliations:** 1 College of Material Science and Art Design, Inner Mongolia Agricultural University, Hohhot 010018, China; ttjjsgh@gmail.com (J.T.);; 2 China Construction Eco-Environmental New Energy Co., Ltd., Nanjing 215000, China

**Keywords:** bamboo, sand barrier, superhydrophobic coating, rutile TiO_2_, epoxy resin, accelerated weathering

## Abstract

Bamboo is a renewable and mechanically robust bio-based material with potential for sand-barrier construction; however, its long-term outdoor use is limited by ultraviolet-induced photoaging, moisture uptake, wind-sand abrasion, and biological colonization. In this study, a fluorine-free EP/TiO_2_/ODTMS superhydrophobic coating was deposited on moso bamboo using a simple spraying process. Rutile TiO_2_ was incorporated as a roughness-building and ultraviolet-shielding filler, waterborne epoxy resin served as a film-forming binder to improve particle anchoring and coating cohesion, and octadecyltrimethoxysilane was used to reduce the surface energy. The formulation containing 50–100 nm rutile TiO_2_ and 2 wt.% epoxy resin provided the best overall balance between surface wettability and mechanical durability, with a water contact angle of 156.4° and a sliding angle of 6.9°. SEM observations revealed a hierarchical surface composed of TiO_2_ particles and microscale agglomerates immobilized within the epoxy matrix. EDS and FTIR results supported the incorporation of TiO_2_- and ODTMS-derived components, while UV–Vis–NIR diffuse-reflectance measurements showed an improved optical response in the ultraviolet region. The coating retained superhydrophobicity after sandpaper abrasion, gravel impact, and tape-peeling tests. After 672 h of xenon-lamp aging, the coated bamboo maintained a water contact angle above 150°, exhibited a total color difference of approximately 7.65, and retained 91.1% of its initial flexural strength. In addition, qualitatively reduced visible mildew colonization was observed during 45 days of high-humidity exposure. These results demonstrate that the spray-deposited coating provides a fluorine-free and potentially scalable approach for improving the water repellency, mechanical durability, and accelerated-weathering resistance of bamboo sand-barrier materials.

## 1. Introduction

Desertification control is essential for ecological restoration in arid and semi-arid regions [1]. Sand barriers can reduce near-surface wind velocity, intercept moving sand and stabilize mobile dunes [2,3,4]. However, traditional biomass barriers are limited by resource availability and short service life, while synthetic barriers may suffer from aging, fragmentation and poor degradability [5,6,7]. In addition, barrier performance is also affected by structural parameters such as opening configuration and porosity distribution [8]. Therefore, bamboo, with rapid renewability, abundant availability and high strength-to-weight ratio, offers a promising bio-based alternative for durable sand barriers, although its hydrophilicity and weathering susceptibility still require effective surface protection [9,10].

Bamboo is a promising bio-based candidate for sand-barrier engineering because of its rapid growth, abundant resources, high strength-to-weight ratio, easy processing and biodegradability. Compared with straw and reed barriers, bamboo possesses higher initial strength and better structural integrity; compared with petroleum-based plastic barriers, bamboo provides better renewability and lower long-term environmental persistence. However, untreated bamboo is not sufficiently durable under harsh desert exposure. Its lignocellulosic structure contains abundant hydroxyl groups and capillary pathways, making it sensitive to water uptake, dimensional instability and fungal colonization. Under strong ultraviolet radiation, lignin-rich surface layers undergo photooxidation, chromophore formation and microcrack development, which can further induce mechanical degradation [11,12]. Long-term weathering can further cause discoloration, surface deterioration and strength loss in bamboo and wood-based materials [13,14]. Wind-driven sand particles further abrade the surface, while intermittent dew, rainfall or humidity accelerates moisture-assisted deterioration. Thus, the key challenge for bamboo sand barriers is not their initial mechanical performance, but the preservation of surface integrity, water repellency and substrate strength during long-term outdoor service.

Bioinspired superhydrophobic modification offers a potential route to improve the durability of lignocellulosic materials. Lotus-leaf self-cleaning and plant water-repellent surfaces provide early biological prototypes for artificial superhydrophobic design [15,16]. Water strider legs, butterfly wings and related artificial surfaces further illustrate how micro/nano-roughness and low-surface-energy chemistry regulate water repellency and adhesion [17,18,19]. The wetting behavior of rough surfaces is commonly interpreted using Young’s equation and the Wenzel and Cassie–Baxter models. In a Cassie-type regime, suitable surface topography allows the liquid interface to bridge across surface asperities, thereby reducing the effective solid–liquid contact fraction [20,21,22,23,24,25]. Stable superhydrophobic behavior therefore requires both appropriate hierarchical topography and low solid–liquid adhesion. In recent years, various superhydrophobic wood and bamboo surfaces have been fabricated through biomimetic surface engineering. Recent reviews summarize the main preparation routes and application scenarios for superhydrophobic wood surfaces [26,27]. Sol–gel and silica-nanoparticle approaches have been used to construct lotus-leaf-like rough structures on wood substrates [28,29]. For bamboo, TiO_2_-film and SiO_2_ soft-lithography coatings have been reported to achieve superhydrophobicity [30,31]. Hydrothermal deposition of TiO_2_ or ZnO can provide inorganic micro/nano-structures and additional UV-shielding or functional protection [32,33,34]. Epoxy-assisted, AKD-based and stain-resistant coatings have also been developed to improve water repellency, dimensional stability, self-cleaning behavior and mechanical durability [35,36,37]. Wax-, cardanol- and bio-based epoxy systems further demonstrate the potential of fluorine-free or multifunctional wood coatings [38,39,40]. Nevertheless, several limitations still restrict their use in sand-barrier environments. Many reported systems focus primarily on initial water repellency while giving insufficient attention to ultraviolet shielding, biological resistance and substrate-strength retention after aging [26,27]. Some coatings rely on fluorinated modifiers or complex template-based fabrication, which may limit their environmental compatibility and large-scale application [30,31,41]. Moreover, weakly bonded nanoparticle layers can be damaged by abrasion, impact or peeling, whereas desert sand barriers must withstand coupled stresses including ultraviolet irradiation, wind-blown sand abrasion, dry–wet cycling, temperature fluctuation and microbial attack [35,42,43]. Therefore, for bamboo sand barriers, a coating should be evaluated as a multifunctional protection system rather than a simple water-repellent layer.

Rutile TiO_2_, waterborne epoxy resin, and octadecyltrimethoxysilane (ODTMS) were selected to provide complementary functions within a spray-deposited coating. Rutile TiO_2_ can contribute to ultraviolet shielding while simultaneously generating particulate surface topography; the cured epoxy matrix can improve coating cohesion and anchor TiO_2_ particles to the bamboo substrate; and ODTMS-derived alkyl chains can reduce the surface energy. However, these functions introduce an inherent structure–property trade-off. Sufficient exposure of TiO_2_ particles is required to maintain hierarchical roughness and a low solid–liquid contact fraction, whereas sufficient epoxy resin is required to prevent particle detachment under abrasion and impact. Excessive epoxy, in contrast, may bury the particulate asperities, reduce surface roughness, and increase droplet pinning. Therefore, the central design challenge is not simply to maximize the initial water contact angle, but to balance surface topography, particle anchoring, water repellency, and mechanical durability.

Accordingly, this study proposes a fluorine-free, spray-deposited EP/TiO_2_/ODTMS coating as a multifunctional protection system for bamboo sand-barrier materials. The scientific novelty lies in the mechanism-guided regulation of TiO_2_ particle size and epoxy-resin content to balance roughness retention and interfacial reinforcement, rather than treating superhydrophobicity as the sole optimization target. The effects of TiO_2_ particle size and epoxy content on surface morphology, wettability, moisture resistance, and mechanical durability were systematically investigated. The optimized coating was further evaluated by SEM, EDS, FTIR, and UV–Vis–NIR diffuse-reflectance spectroscopy, together with time-dependent contact-angle measurements, water-absorption tests, sandpaper abrasion, gravel impact, tape peeling, xenon-lamp aging, flexural-strength testing, and qualitative mildew-growth observation under high-humidity exposure. This work aims to establish a practical coating-design strategy for improving the water repellency, mechanical robustness, and accelerated-weathering resistance of bamboo sand barriers.

## 2. Results and Discussion

### 2.1. Morphological Construction of a Hierarchical Rough Coating

**Figure 1:**
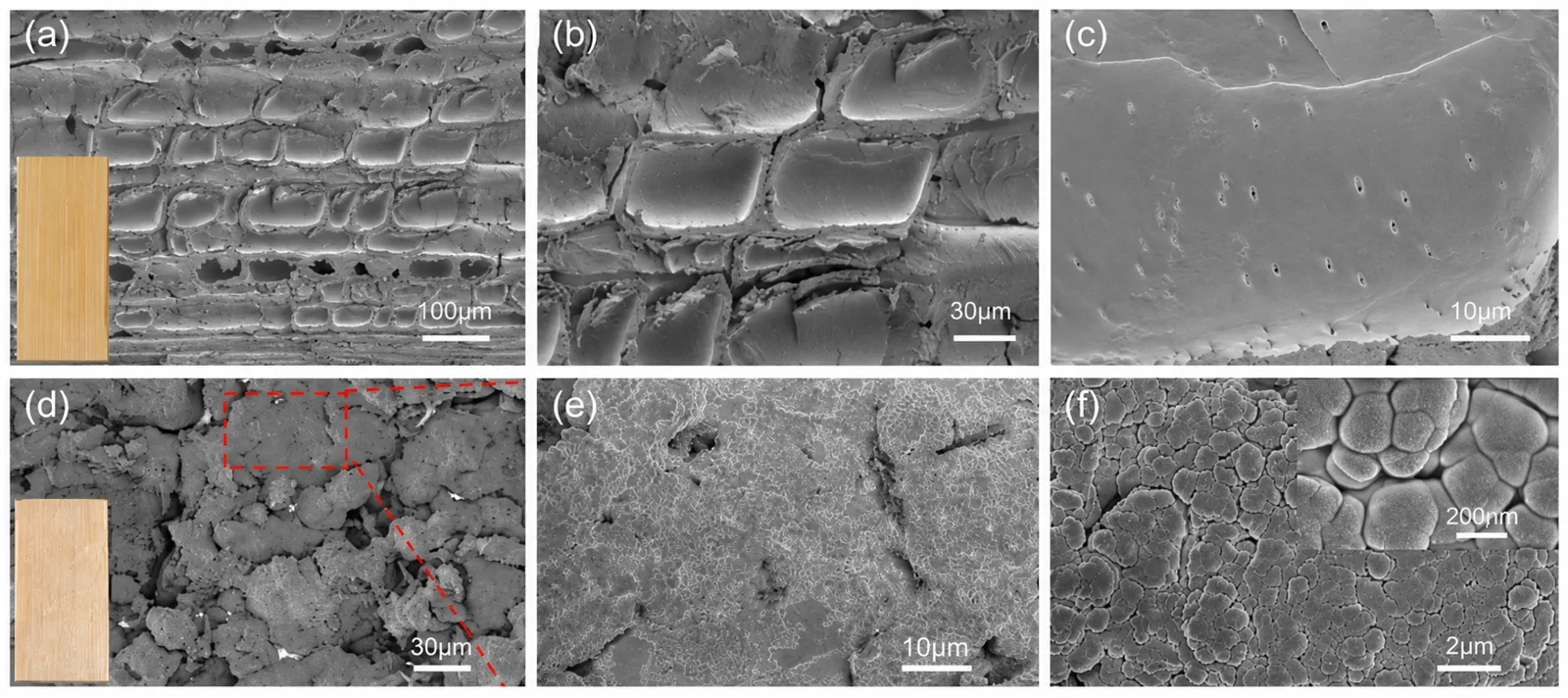
SEM images of untreated bamboo and EP/TiO_2_/ODTMS-coated bamboo at different magnifications: (**a**–**c**) untreated bamboo; (**d**–**f**) EP/TiO_2_/ODTMS-coated bamboo. The red dashed rectangle in (**d**) indicates the region shown at higher magnification in (**e**).

As shown in Figure 1, SEM observations revealed a continuous coating layer on the bamboo surface after spray deposition. Unlike the relatively smooth untreated bamboo surface (Figure 1a–c), the coated surface was covered by microscale agglomerates and nanoscale TiO_2_ particles (Figure 1d–f). During spraying and curing, TiO_2_ particles and epoxy prepolymer co-deposited onto the substrate; after curing, the epoxy network acted as an adhesive framework that immobilized the TiO_2_ particles while leaving part of the particulate texture exposed. This architecture is important because the exposed TiO_2_ particles provide hierarchical surface topography and reduce the effective solid–liquid contact fraction, whereas the epoxy matrix improves particle anchoring and limits particle loss during abrasion or impact.

**Figure 2:**
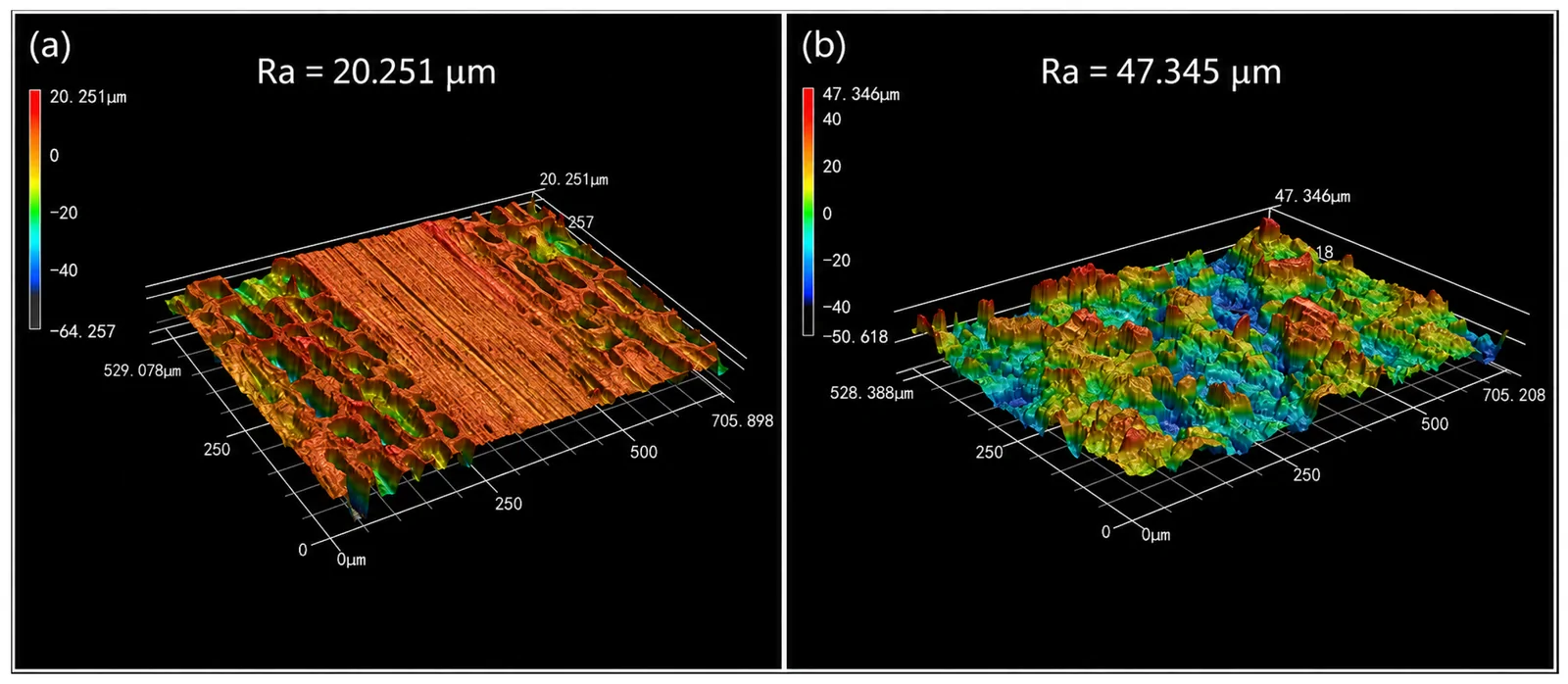
Three-dimensional laser-confocal topographies of (**a**) untreated bamboo and (**b**) EP/TiO_2_/ODTMS-coated bamboo.

As shown in Figure 2, laser confocal microscopy further confirmed that coating deposition increased the surface roughness. The untreated bamboo and EP/TiO_2_/ODTMS-coated bamboo exhibited arithmetic average roughness (Ra) values of 20.251 and 47.345 μm, respectively (Figure 2a,b). The approximately 2.34-fold increase in Ra demonstrates that the deposited coating formed a more pronounced hierarchical surface topography. This topography is consistent with wetting behavior in which the liquid interface bridges across surface asperities, thereby reducing the effective solid–liquid contact fraction. Meanwhile, the immobilization of the particulate structure within the epoxy-containing matrix helps limit the formation of loosely attached agglomerates that could become weak points during repeated gravel impact.

### 2.2. Chemical Composition and Interfacial Architecture

**Figure 3:**
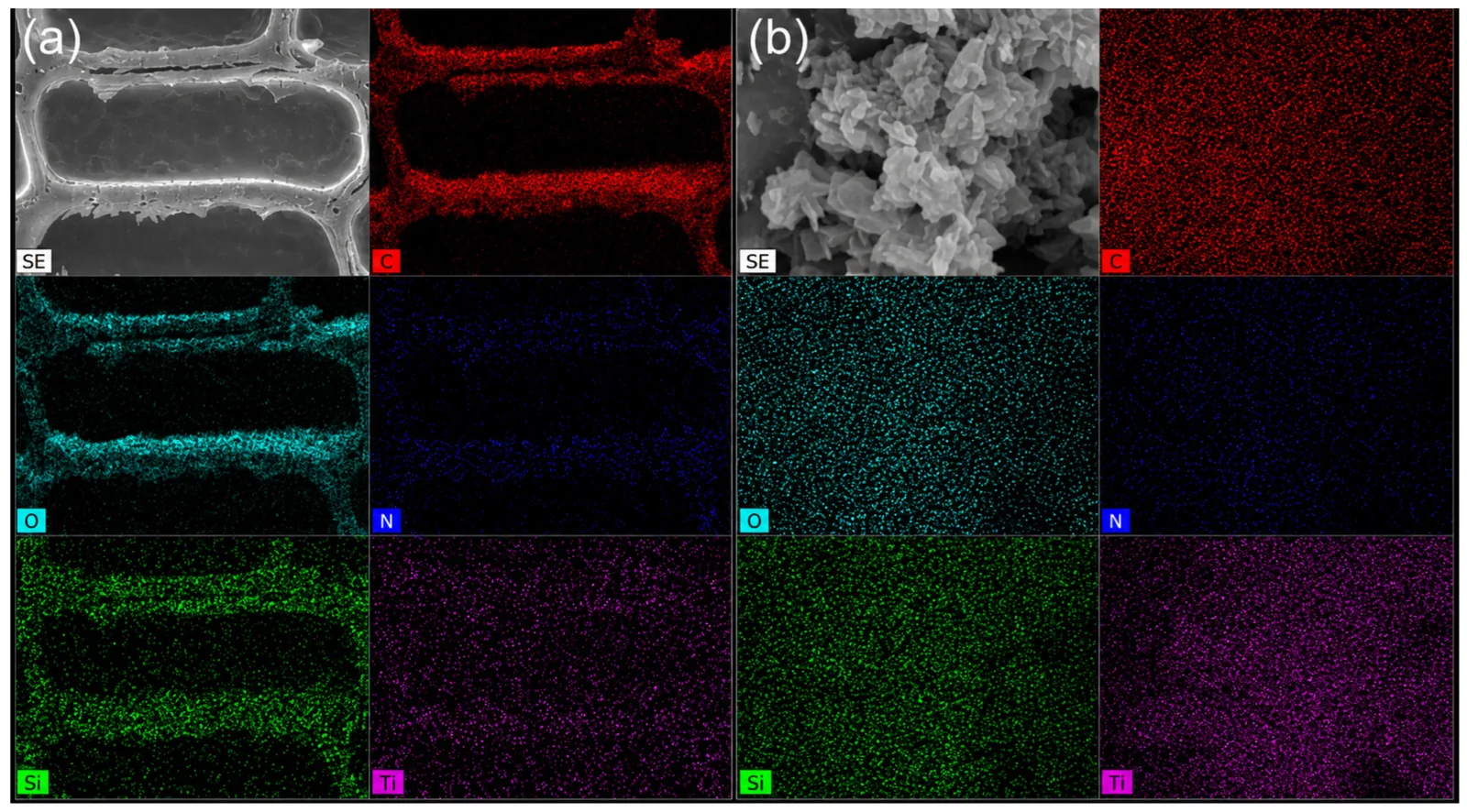
Representative SEM images and corresponding EDS elemental mappings of untreated bamboo and EP/TiO_2_/ODTMS-coated bamboo: (**a**) untreated bamboo; (**b**) EP/TiO_2_/ODTMS-coated bamboo. The elemental maps show the distributions of C, O, N, Si, and Ti.

As shown in Figure 3, the untreated bamboo surface was dominated by C and O signals, whereas the EP/TiO_2_/ODTMS-coated surface exhibited broadly distributed C, O, Si, and Ti signals. The Ti and Si signals are consistent with the presence of TiO_2_ and ODTMS-derived components, respectively (Figure 3b). Compared with untreated bamboo, the coated surface contained 33.29 wt.% Ti and 2.58 wt.% Si, while no Ti and only 0.15 wt.% Si were detected on the untreated surface (Table 1). The broadly distributed Ti- and Si-containing signals indicate that these coating components were present throughout the analyzed surface area rather than being confined to isolated regions.

**Figure 4:**
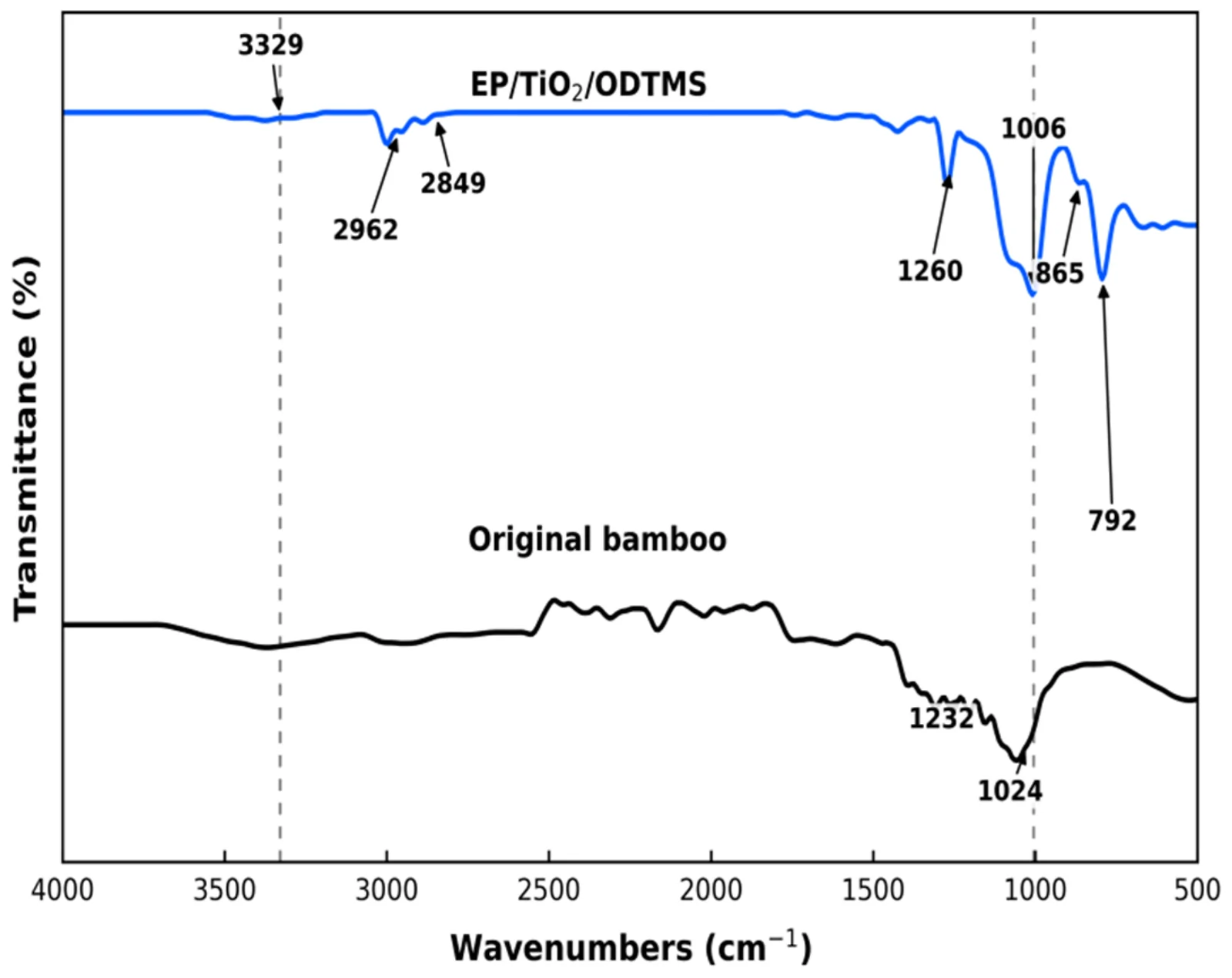
FTIR spectra of untreated bamboo and EP/TiO_2_/ODTMS-coated bamboo.

As shown in Figure 4, the FTIR spectrum of the EP/TiO_2_/ODTMS-coated bamboo differed from that of untreated bamboo, indicating changes in the surface composition after coating deposition. The peaks near 2962 and 2849 cm^−1^ were assigned to the C–H stretching vibrations of terminal methyl and methylene groups in the long alkyl chains, supporting the presence of ODTMS-derived organic components in the coating [44]. The spectral changes in the 1000–1150 cm^−1^ region, including the feature near 1006 cm^−1^, are consistent with Si–O–Si vibrations associated with siloxane condensation; however, this region also overlaps with the C–O vibrations of bamboo components [44,45]. The signal near 1260 cm^−1^ was therefore regarded as a coating-related spectral change rather than being assigned to a unique chemical bond. The attenuation of the broad hydroxyl-related band near 3329 cm^−1^ may be associated with surface coverage by the composite coating and the reduced contribution of exposed bamboo hydroxyl groups. These spectral changes support the presence of ODTMS-derived components but do not unambiguously demonstrate the formation of Si–O–C covalent bonds with the bamboo surface.

### 2.3. UV-Shielding Capability

**Figure 5:**
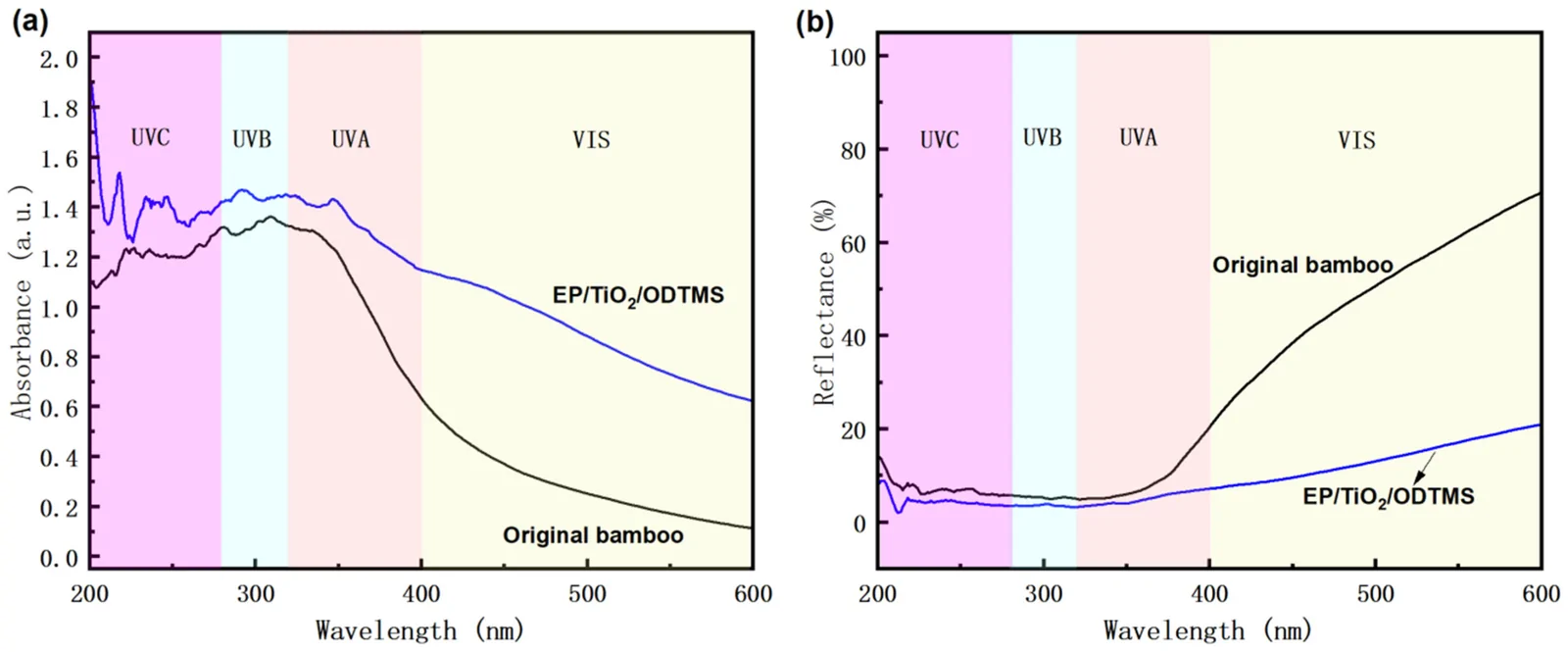
UV-Vis-NIR spectra of untreated bamboo and EP/TiO_2_/ODTMS-coated bamboo: (**a**) absorbance spectra; (**b**) reflectance spectra.

As shown in Figure 5, the EP/TiO_2_/ODTMS-coated bamboo exhibited a markedly different ultraviolet optical response from untreated bamboo. Untreated bamboo showed relatively limited reflectance in the ultraviolet region, whereas the coated bamboo exhibited enhanced ultraviolet absorbance and reflectance. This result indicates that the composite coating reduced the transmission of high-energy ultraviolet radiation toward the bamboo surface. The enhanced optical response may arise from the combined effects of the complete coating, with rutile TiO_2_ likely contributing through ultraviolet absorption, scattering, and reflection. Similar TiO_2_-based inorganic structures have been reported to improve the ultraviolet-protective performance of wood surfaces [33]. However, because a structurally matched TiO_2_-free coating was not included in the present study, the independent contribution of TiO_2_ could not be quantitatively separated from that of the epoxy/ODTMS-containing matrix. Therefore, Figure 5 demonstrates the ultraviolet-shielding response of the complete EP/TiO_2_/ODTMS coating rather than the isolated effect of TiO_2_.

### 2.4. Optimization of Wetting Performance: TiO_2_ Particle Size and Epoxy-Resin Content

As summarized in Table 2, the wetting behavior of the coatings depended on both TiO_2_ particle size and epoxy-resin content. With a fixed particle size, increasing epoxy content generally reduced hydrophobicity because excessive resin filled the hierarchical roughness and buried the modified TiO_2_ asperities. Nevertheless, insufficient resin weakened particle adhesion. The formulation containing 50–100 nm rutile TiO_2_ and 2 wt.% epoxy resin was selected as the best overall formulation rather than the formulation with the highest initial WCA. Although the epoxy-free coating showed a slightly higher WCA and lower SA, the absence of a binder resulted in weaker particle anchoring. The addition of 2 wt.% epoxy retained a high WCA of 156.4° and an SA below 10°, while providing improved coating cohesion and mechanical durability. At higher epoxy contents, the resin increasingly covered the particulate surface texture and increased droplet pinning. Smaller particles can generate high initial WCA but may pack too densely or be more easily buried, whereas larger particles form roughness with higher droplet pinning and poorer sliding behavior.

### 2.5. Water Repellency and Moisture Uptake

**Figure 6:**
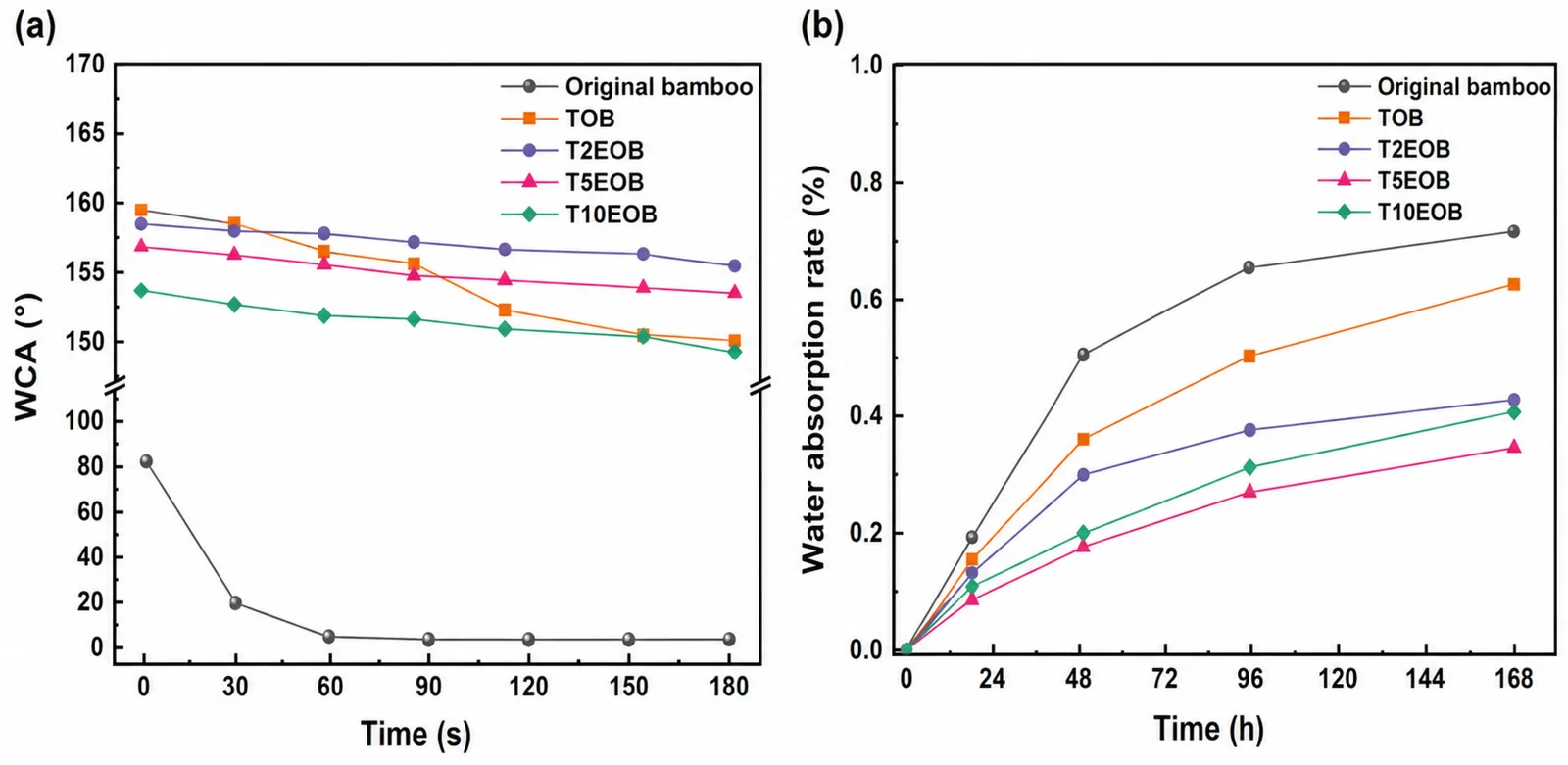
Time-dependent water contact angle and water absorption of original bamboo and bamboo coated with different epoxy contents: (**a**) variation in the water contact angle of original bamboo, TOB, T2EOB, T5EOB, and T10EOB during 180 s; (**b**) water absorption of original bamboo and coated bamboo samples during 168 h of immersion.

The sample codes and corresponding epoxy contents used for the water-repellency comparison are summarized in Table 3. As shown in Figure 6a, the coated bamboo samples retained relatively high water contact angles throughout the 180 s measurement period, although their contact angles gradually decreased with time. This time-dependent decrease indicates that the coatings delayed rapid liquid penetration but did not completely prevent progressive wetting. In contrast, original bamboo exhibited rapid water spreading and absorption because of its exposed hydrophilic groups and capillary pathways.

The 168 h immersion results further demonstrate the moisture-barrier effect of the coatings (Figure 6b). Original bamboo exhibited substantially greater water absorption than the coated samples, whereas the epoxy-containing coatings suppressed moisture uptake more effectively. Among the tested formulations, the coating with moderate epoxy content provided a favorable balance between preserving the hydrophobic particulate texture and forming a continuous polymer barrier. The reduced water uptake can be attributed to the combined effects of the low-surface-energy ODTMS-derived components, the hierarchical TiO_2_-based surface structure, and the physical barrier provided by the cured epoxy network.

### 2.6. Mechanical Robustness Under Simulated Sand-Barrier Stresses

**Figure 7:**
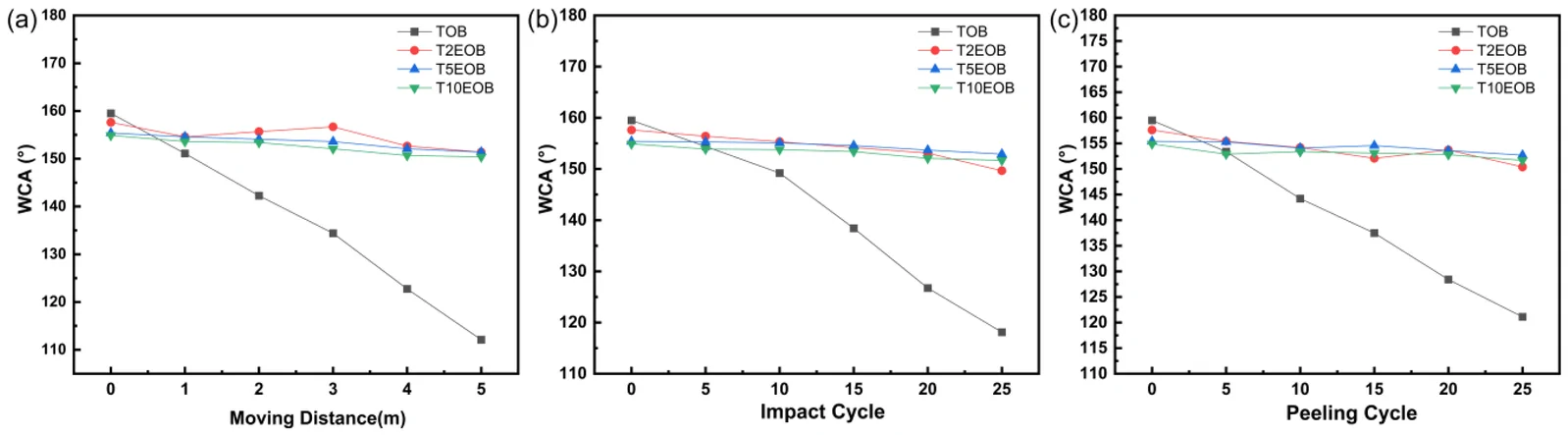
Changes in the water contact angle of EP/TiO_2_/ODTMS-coated bamboo during (**a**) sandpaper abrasion, (**b**) gravel impact, and (**c**) tape peeling.

Mechanical robustness is essential for sand barriers because wind-driven particles, installation handling and repeated surface contact can rapidly destroy fragile superhydrophobic textures. Previous studies have shown that durable superhydrophobic wood coatings require not only micro/nano-roughness and low surface energy but also sufficient interfacial adhesion and cohesive strength to resist abrasion or peeling [35,39,43]. As shown in Figure 7, the EP/TiO_2_/ODTMS coating was evaluated under three representative mechanical stresses, including sandpaper abrasion, gravel impact, and tape peeling, to simulate damage conditions relevant to bamboo sand-barrier service. Without epoxy, the particle layer was mainly attached by weak physical interactions and the WCA declined sharply after mechanical damage. Moderate epoxy contents retained WCA values above the superhydrophobic threshold after abrasion, impact and peeling because the cured resin network distributed external stress and prevented large-scale detachment of TiO_2_ particles. However, excessive epoxy reduced roughness and did not provide proportional durability gains. Thus, the data establish a trade-off: enough epoxy is required for adhesion and cohesion, but too much epoxy compromises surface roughness and droplet mobility. The quantitative changes in WCA after the prescribed abrasion distances and impact or peeling cycles are presented in Figure 7. Supplementary Figure S1 provides representative photographs of the experimental apparatus, specimen arrangement, and selected operating stages. These photographs are intended to illustrate the test configuration rather than to represent a continuous recording of the complete test cycles.

### 2.7. Accelerated Weathering: Color Stability, Wettability Retention and Flexural Protection

**Figure 8:**
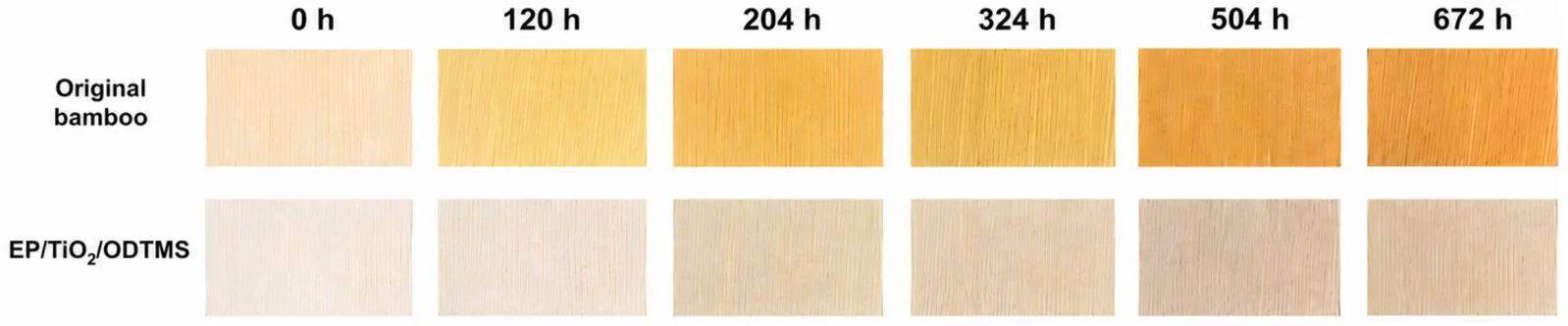
Macroscopic appearance of bamboo samples during xenon-lamp aging.

**Figure 9:**
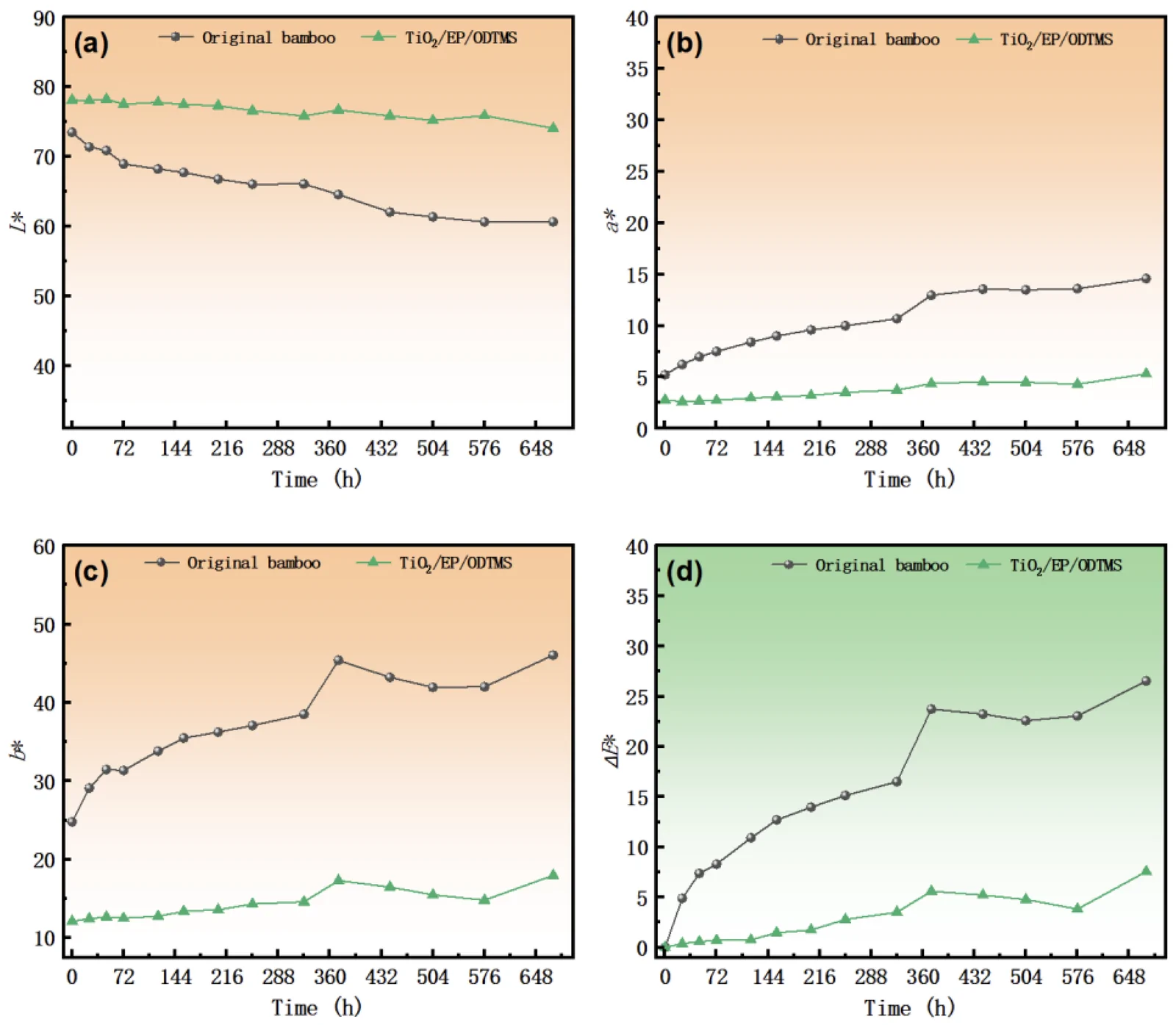
Changes in (**a**) L*, (**b**) a*, (**c**) b*, and (**d**) ΔE* values of untreated bamboo and EP/TiO_2_/ODTMS-coated bamboo during 672 h of xenon-lamp aging.

As shown in Figure 8, accelerated xenon-lamp aging caused pronounced yellowing and browning of untreated bamboo, whereas the EP/TiO_2_/ODTMS-coated bamboo retained a substantially lighter appearance. Weathering-induced discoloration of bamboo and wood is commonly associated with ultraviolet-driven changes in lignin and other lignocellulosic components [13,14]. The color-coordinate results further quantified this difference (Figure 9). After 672 h of xenon-lamp aging, the EP/TiO_2_/ODTMS-coated bamboo exhibited a total color difference (ΔE) of approximately 7.65, which was substantially lower than that of untreated bamboo. This lower ΔE value indicates that the coating improved the color stability of the bamboo surface. Rutile TiO_2_ may contribute through ultraviolet attenuation, while the epoxy/ODTMS-containing layer may reduce the access of moisture and oxygen to the substrate. However, because a compositionally matched TiO_2_-free coating was not tested, the independent contribution of rutile TiO_2_ cannot be quantitatively distinguished from that of the complete composite coating.

**Figure 10:**
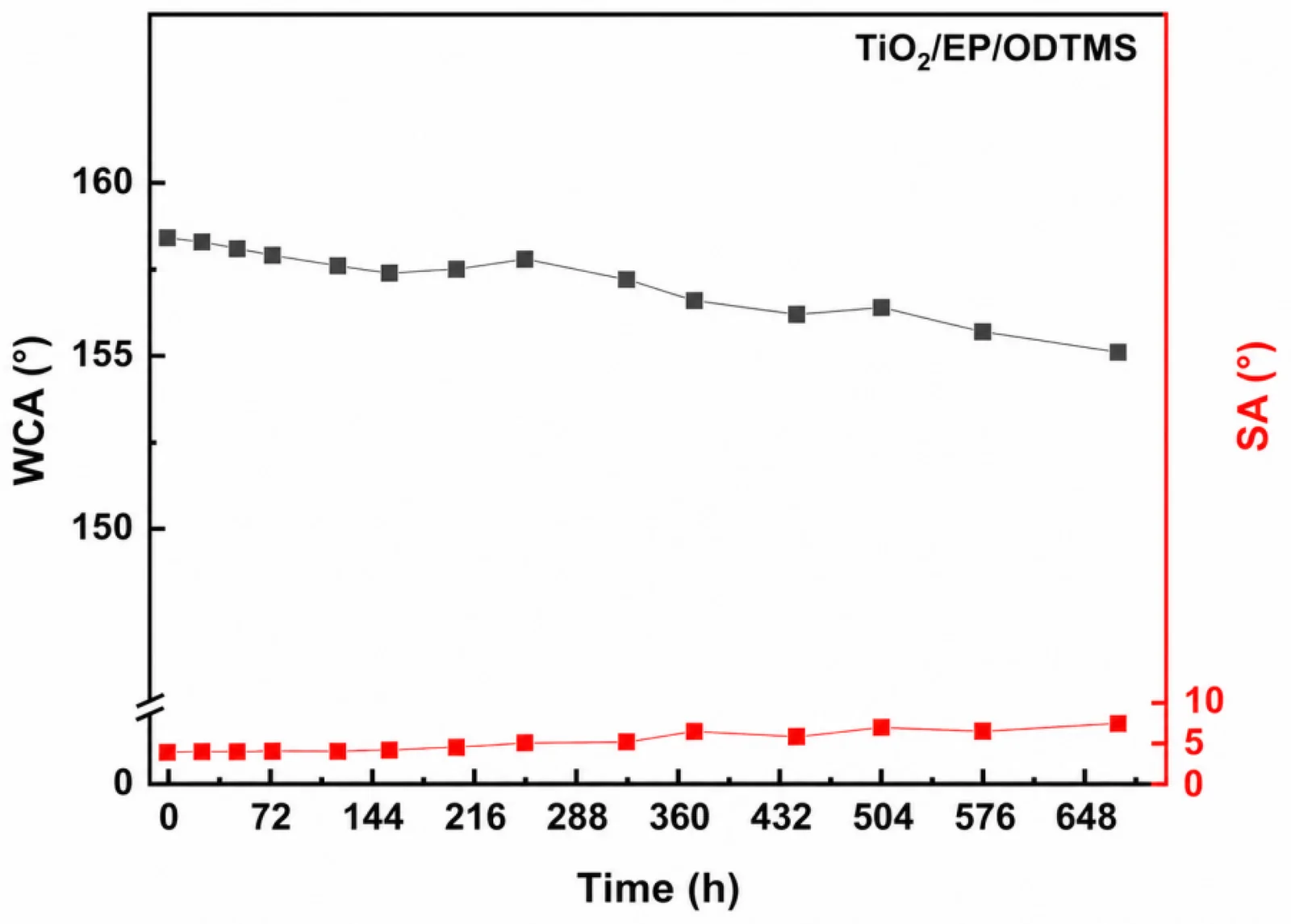
WCA and SA retention of EP/TiO_2_/ODTMS-coated bamboo during 672 h xenon-lamp aging.

The wetting properties of the coated bamboo were also largely retained during accelerated aging. As shown in Figure 10, the water contact angle remained above 150° throughout the 672 h test, while the sliding angle remained below 10°. This retention may be associated with the combined effects of ultraviolet attenuation by the composite coating, the presence of low-surface-energy ODTMS-derived groups, and stabilization of the particulate surface structure by the epoxy-containing matrix. The flexural-strength results are summarized in Table 4. After 672 h of aging, untreated bamboo retained 78.7% of its initial flexural strength, whereas the EP/TiO_2_/ODTMS-coated bamboo retained 91.1%. These results indicate that the complete composite coating reduced the deterioration of the bamboo substrate under the applied accelerated-aging conditions, potentially through reduced ultraviolet exposure, suppressed moisture ingress, and physical isolation of the bamboo surface.

### 2.8. Qualitative Mildew-Growth Suppression Under High-Humidity Exposure

**Figure 11:**
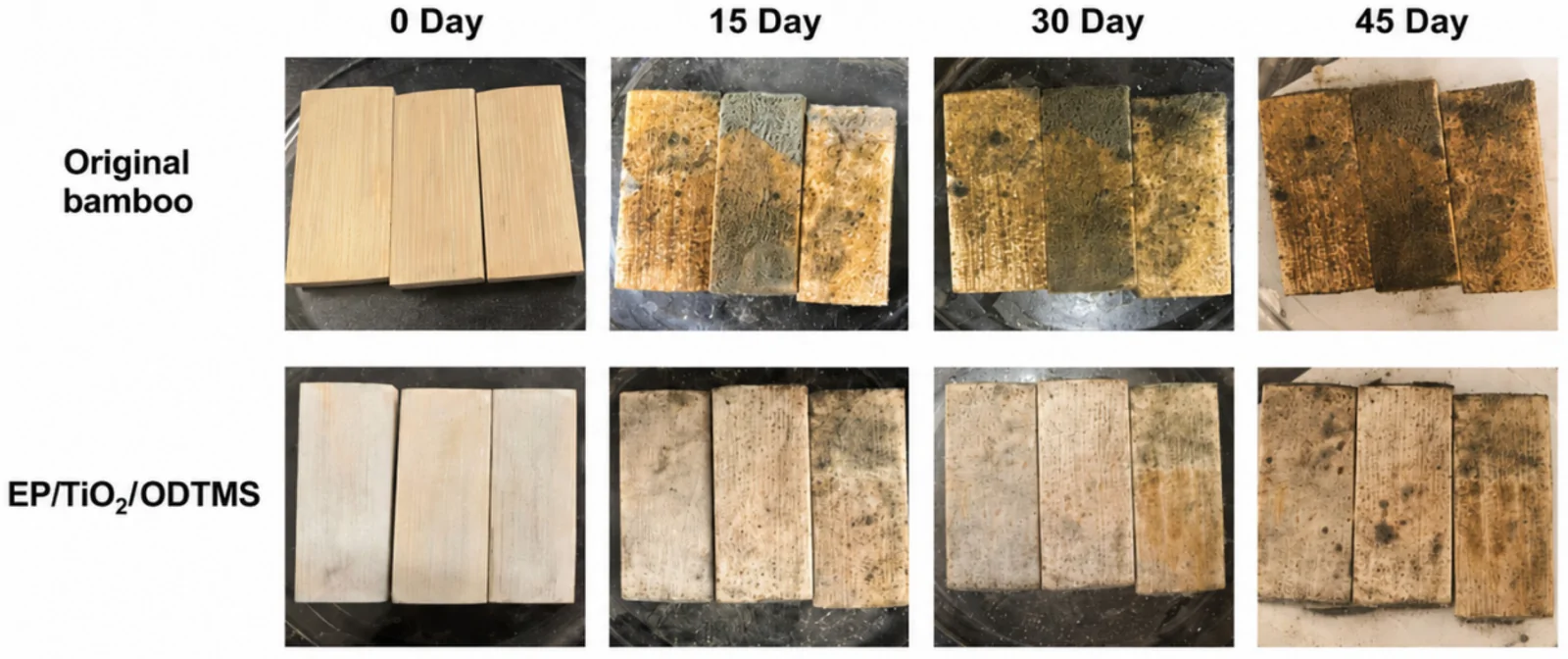
Representative photographs of visible mildew colonization on untreated bamboo and EP/TiO_2_/ODTMS-coated bamboo after 0, 15, 30, and 45 days of high-humidity exposure.

As shown in Figure 11, untreated bamboo exhibited progressively increasing visible mildew colonization during the 45-day high-humidity exposure, whereas the EP/TiO_2_/ODTMS-coated bamboo showed substantially less visible colonization over the same period. The extensive mildew development on untreated bamboo may be associated with its hydrophilic surface, open capillary pathways, and the availability of cellulose and hemicellulose as nutrient sources. Fungal growth on lignocellulosic materials is strongly influenced by moisture availability, oxygen supply, and access to organic nutrients [46,47].

The reduced visible mildew colonization on the coated bamboo may be associated with lower liquid-water retention and reduced moisture transport toward the underlying substrate. The low-surface-energy ODTMS-derived components and hierarchical surface topography may limit the formation of a continuous water film, while the epoxy-containing layer provides an additional physical barrier to moisture penetration. These effects may create surface conditions that are less favorable for visible mildew development.

However, no standardized fungal strain was inoculated, and fungal coverage, colony number, spore germination, and microbial viability were not quantitatively measured. Therefore, Figure 11 provides qualitative evidence of reduced visible mildew colonization rather than confirmation of antifungal or fungicidal activity. The specific biological mechanism responsible for this observation cannot be conclusively determined from the present experiment.

### 2.9. Mechanistic Interpretation and Engineering Relevance

The overall performance of the EP/TiO_2_/ODTMS coating can be interpreted as a multi-barrier protection mechanism involving reduced water uptake, ultraviolet attenuation, and mechanical stabilization. These effects are coupled through the hierarchical particulate structure and the epoxy-containing matrix.

First, the rutile TiO_2_ particles act as an inorganic roughness-building component. The resulting hierarchical surface structure enables the liquid interface to bridge across surface asperities, thereby reducing the effective solid–liquid contact fraction and producing wetting behavior consistent with a Cassie-type regime [22,23,25]. Rutile TiO_2_ may also contribute to the ultraviolet-shielding response of the complete coating through absorption, scattering, and reflection of ultraviolet radiation [33]. However, because a compositionally matched TiO_2_-free coating was not evaluated, the independent ultraviolet-protective contribution of TiO_2_ cannot be quantitatively distinguished from that of the epoxy/ODTMS-containing matrix.

Second, the ODTMS-derived long alkyl chains reduce the surface energy and solid–liquid adhesion of the coating. The FTIR results are consistent with the presence of ODTMS-derived organic components and siloxane species, although they do not unambiguously demonstrate the formation of covalent Si–O–C bonds with the bamboo substrate. The resulting low-surface-energy surface promotes droplet mobility and reduces liquid-water retention.

Third, the cured epoxy-containing matrix provides structural reinforcement by anchoring the TiO_2_ particles and improving coating cohesion. This reinforcement limits large-scale particle detachment during sandpaper abrasion, gravel impact, and tape peeling. Nevertheless, excessive epoxy resin can cover the particulate asperities, reduce the effective surface roughness, and increase droplet pinning. The optimized epoxy content therefore reflects a balance between maintaining the hierarchical surface texture and improving mechanical stability.

Taken together, the EP/TiO_2_/ODTMS coating functions as a multifunctional protective layer under the tested laboratory conditions. The coating combines water repellency, mechanical durability, moisture-barrier performance, and resistance to accelerated xenon-lamp aging. Further outdoor exposure studies are required to determine its long-term performance under actual desert service conditions.

## 3. Materials and Methods

### 3.1. Materials

Moso bamboo (*Phyllostachys edulis*) culms, aged 3–5 years, were obtained from Wuhu, Anhui Province, China. The culms were machined into rectangular specimens with dimensions of 50 mm × 20 mm × 5 mm for coating preparation, wettability measurements, water-absorption tests, xenon-lamp aging and flexural testing. Before coating, the bamboo specimens were polished to remove surface impurities, washed with absolute ethanol and deionized water, and dried at 60 °C. Unless otherwise specified, the specimens were conditioned at 20 ± 2 °C and 65 ± 5% relative humidity before testing.

Octadecyltrimethoxysilane (ODTMS, C_21_H_46_O_3_Si, ≥90%) was purchased from Shanghai Aladdin Biochemical Technology Co., Ltd., Shanghai, China. Rutile TiO_2_ powders with particle sizes of 20 nm, 50–100 nm and 1 μm were supplied by Nangong Xindun Alloy Welding Material Spraying Co., Ltd., Nangong, China. Waterborne epoxy resin AB adhesive (JH-5560) was obtained from Hangzhou Wuhuigang Adhesive Co., Ltd., Hangzhou, China. Ethyl acetate and absolute ethanol were of analytical grade and used as received. Deionized water was used throughout the experiments.

### 3.2. Preparation of EP/TiO_2_/ODTMS-Coated Bamboo

The epoxy prepolymer was prepared by mixing component A of the waterborne epoxy resin with curing agent B at a mass ratio of 2:1 under continuous stirring. To prepare the coating suspension, 0.5 g of rutile TiO_2_ powder was dispersed in 20 mL of ethyl acetate and ultrasonicated for 30 min. Subsequently, 1 mL of deionized water and 1 mL of ODTMS were added to promote the hydrolysis of ODTMS and the surface modification of TiO_2_ particles. After stirring for 30 min, the epoxy prepolymer was introduced at designed mass fractions of 0, 1, 2, 5, 10 and 15 wt.%, followed by continuous stirring for 1 h to obtain a homogeneous EP/TiO_2_/ODTMS spraying suspension.

The prepared suspension was sprayed onto the bamboo surface using an electric spray pen. The spraying pressure was maintained at 20 kPa, and the distance between the nozzle and the bamboo surface was controlled at 15 cm. Each specimen was sprayed for three passes, with an interval of approximately 5 min between successive passes to allow partial solvent evaporation. The coated specimens were then cured at 60 °C for 6 h to obtain EP/TiO_2_/ODTMS-coated bamboo.

### 3.3. Surface Morphology and Chemical Characterization

Unless otherwise stated, the optimized formulation containing 50–100 nm rutile TiO_2_ and 2 wt.% epoxy resin was used for subsequent characterization. For SEM observation, bamboo specimens with dimensions of approximately 5 mm × 5 mm × 2 mm were mounted on conductive stubs using conductive adhesive and sputter-coated with platinum. The surface morphology of untreated and EP/TiO_2_/ODTMS-coated bamboo was observed using a scanning electron microscope (S-4800, Hitachi, Tokyo, Japan) at an accelerating voltage of 3.0 kV. Energy-dispersive spectroscopy (EDS) attached to the SEM was used to analyze the surface elemental composition and distribution of untreated and EP/TiO_2_/ODTMS-coated bamboo. Three-dimensional surface topography and arithmetic average roughness were measured using a laser confocal scanning microscope (VK-X1000, Keyence, Osaka, Japan).

Fourier-transform infrared spectroscopy (FTIR, Nicolet iS20, Thermo Fisher Scientific, Waltham, MA, USA) was used to analyze surface chemical structures in the range of 4000–500 cm^−1^. UV–Vis–NIR diffuse reflectance spectra were recorded using a UV/visible/near-infrared spectrophotometer (UH4150, Hitachi, Japan) to evaluate the ultraviolet-shielding ability of the coating.

### 3.4. Wettability and Water-Resistance Measurements

The surface wettability was evaluated using a contact-angle measuring instrument (PZ-350SD, Beijing Pinzhi Chuangsi Precision Instrument Co., Ltd., Beijing, China). A 5 μL deionized water droplet was placed on the bamboo surface, and the static water contact angle (WCA) was calculated from the droplet profile. The sliding angle (SA) was measured by placing a 10 μL deionized-water droplet on the coated surface and tilting the sample stage at approximately 1°s^−1^ using the instrument-controlled sliding-angle program. The angle at which the droplet began to move continuously was recorded as the SA. Five measurements were performed at different positions on each specimen, and the results are reported as mean ± standard deviation.

The time-dependent WCA was recorded for 180 s at 30 s intervals. Water absorption was measured by immersing the specimens in deionized water for different periods. Water absorption was determined after 12, 24, 48, 96, and 168 h of immersion, with the value at 0 h defined as zero. At each measurement time, the specimens were removed from the water, gently wiped to remove surface water, and immediately weighed. The water absorption was calculated according to Equation (1):

(1) $$W\;=\;\frac{m_{t}\;-{\;m}_{0}}{m_{0}}\;\times \;100\%$$

where $m_{0}$ is the initial dry mass of the specimen and $m_{t}$ is the mass of the specimen after immersion for a given time.

### 3.5. Mechanical Durability Tests

These experiments were conducted as comparative laboratory durability evaluations adapted from established procedures reported for superhydrophobic wood and coating systems [48,49]. The sandpaper-abrasion procedure followed a published protocol using 1000-grit sandpaper, a 100 g applied load, defined abrasion distances, and subsequent WCA and SA measurements [48]. The gravel-impact test was designed with reference to previously reported falling-particle evaluations and the general falling-abrasive principle described in ASTM D968-25 [50]. Because the substrate, abrasive material, movement pattern, impact conditions, and evaluation endpoints were adapted for bamboo sand-barrier applications, the experiments do not claim full compliance with ASTM D968-25.

The mechanical durability of the coating was evaluated using sandpaper-abrasion, tape-peeling, and gravel-impact tests to simulate mechanical damage during sand-barrier service. In the sandpaper-abrasion test, the coated surface was placed face-down on 1000-grit sandpaper under a 100 g load. The specimen was first moved 10 cm along the longitudinal direction of the bamboo and then 10 cm in the perpendicular direction. This procedure was defined as one abrasion cycle, corresponding to a total abrasion distance of 20 cm. The WCA and SA were measured after every five abrasion cycles, corresponding to a cumulative abrasion distance of 1 m. A total of 25 cycles, corresponding to a cumulative abrasion distance of 5 m, were performed.

In the tape-peeling test, an 18 mm-wide 3M transparent adhesive tape(3M Company, St. Paul, MN, USA) was firmly attached to the coated surface and rolled back and forth three times under a 200 g load to ensure sufficient contact. The tape was then rapidly peeled from the surface at an angle of approximately 90°. The WCA and SA were measured after every five peeling cycles, and a total of 25 peeling cycles were conducted.

The repeated tape-peeling procedure was adapted from established mechanical-durability evaluations used for superhydrophobic wood surfaces [49]. In the gravel-impact test, gravel collected from the Ulan Buh Desert and screened through a 40-mesh sieve was used. The specimen was fixed at an inclination angle of 45°, and 150 g of gravel was released through a funnel from a vertical height of 40 cm above the coated surface. The WCA and SA were measured after every five impact cycles, and a total of 25 impact cycles were conducted. Representative photographs of the apparatus, specimen arrangement, and selected operating stages of the sandpaper-abrasion and gravel-impact tests are provided in Supplementary Figure S1. The mechanical-durability assessment is based on the WCA values measured after the predefined abrasion distances and impact or peeling cycles shown in Figure 7.

### 3.6. Accelerated Weathering and Color Measurement

Accelerated weathering was performed in a xenon-lamp aging chamber (DES-Gmn7320-HL, Shenzhen Desheng Instrument Technology Co., Ltd., Shenzhen, China) under continuous xenon-arc irradiation over the wavelength range of 200–1100 nm. The irradiance was maintained at 550 W m^−2^, the black-panel temperature at 55 ± 3 °C, the chamber temperature at 50 ± 3 °C, and the relative humidity at 20 ± 5%. No water-spray or condensation cycle was applied. The total exposure duration was 672 h.

Surface color parameters were measured using a precision colorimeter (WR-10, Shenzhen Wave Optoelectronics Technology Co., Ltd., Shenzhen, China) based on the CIE Lab* color system. The total color difference was calculated according to Equation (2):

(2) $$\Delta E\;=\;{\left[\;(\Delta L^{*}{)}^{2}\;+\;(\Delta a^{*}{)}^{2}+\;(\Delta b^{*}{)}^{2}\;\right]}^{1/2}$$

where $\Delta L^{*}$, $\Delta a^{*}$ and $\Delta b^{*}$ are the differences in L*, $a^{*}$ and $b^{*}$ values between the aged and initial specimens, respectively.

### 3.7. Flexural Testing

Flexural properties were measured using a computer-controlled electronic universal testing machine (WDW-10, Jinan Tianchen Testing Machine Manufacturing Co., Ltd., Jinan, China). Three-point bending tests were conducted with a span of 40 mm and a loading rate of 2 mm/min. At least three valid specimens were tested for each group.

The flexural strength $\sigma_{b}$ was calculated according to Equation (3):

(3) $$\sigma_{b}\;=\;\frac{3P_{\max}L}{2bh^{2}}$$

where $P_{\max}$ is the maximum load, L is the span, *b* is the specimen width and h is the specimen thickness. The flexural-strength retention rate after aging was calculated by comparing the flexural strength after 672 h of xenon-lamp aging with the initial flexural strength before aging.

### 3.8. High-Humidity Mildew-Exposure Test

Untreated and EP/TiO_2_/ODTMS-coated bamboo specimens were cleaned and placed in a closed humid chamber at 25 ± 2 °C and 90 ± 5% relative humidity for 45 days. Surface appearance and visible mildew colonization were recorded after 0, 15, 30, and 45 days. Visible mildew colonization was qualitatively compared between untreated and coated bamboo specimens. No standardized fungal inoculation or quantitative microbial analysis was performed.

## 4. Conclusions

In this study, a fluorine-free, spray-deposited EP/TiO_2_/ODTMS superhydrophobic coating was developed to improve the water repellency, mechanical durability, and accelerated-weathering resistance of bamboo sand-barrier materials. The optimized formulation containing 50–100 nm rutile TiO_2_ and 2 wt.% epoxy resin produced a hierarchical micro/nano-textured surface with a water contact angle of 156.4° and a sliding angle of 6.9°. SEM and laser-confocal observations confirmed the formation of a particulate hierarchical surface. EDS analysis detected broadly distributed Ti and Si signals on the coated surface, while FTIR spectra supported the presence of ODTMS-derived alkyl and siloxane components. These results support the successful incorporation of TiO_2_- and ODTMS-related components into the deposited coating, although they do not unambiguously demonstrate the formation of covalent Si–O–C bonds with the bamboo substrate.

UV-Vis-NIR diffuse-reflectance measurements demonstrated an enhanced ultraviolet optical response of the complete EP/TiO_2_/ODTMS coating. Because a compositionally matched TiO_2_-free coating was not evaluated, the independent contribution of rutile TiO_2_ could not be quantitatively distinguished from that of the epoxy/ODTMS-containing matrix. The optimized coating retained superhydrophobic wetting characteristics after sandpaper abrasion, gravel impact, and tape peeling, indicating that an appropriate epoxy content improved particle anchoring without completely covering the hierarchical surface texture.

After 672 h of xenon-lamp aging, the coated bamboo maintained a water contact angle above 150°, exhibited a total color difference of approximately 7.65, and retained 91.1% of its initial flexural strength, compared with 78.7% for untreated bamboo. The coated bamboo also exhibited less visible mildew colonization during 45 days of high-humidity exposure. Because no standardized fungal inoculation or quantitative microbial analysis was conducted, this observation should be interpreted as qualitative evidence of reduced visible mildew colonization rather than confirmation of antifungal or fungicidal activity. Overall, the EP/TiO_2_/ODTMS coating provides a fluorine-free and potentially scalable strategy for improving the water repellency, mechanical durability, moisture resistance, and accelerated-weathering performance of bamboo sand-barrier materials under the tested laboratory conditions. Further outdoor exposure studies are required to evaluate its long-term performance under actual desert service conditions.

## Figures and Tables

**Table 1:** EDS elemental compositions of untreated and EP/TiO_2_/ODTMS-coated bamboo surfaces.

Sample	Basis	C (%)	O (%)	N (%)	Si (%)	Ti (%)
Untreated bamboo	wt.%	50.45	49.41	0	0.15	0
	at.%	43.36	56.57	0	0.07	0
EP/TiO_2_/ODTMS-coated bamboo	wt.%	29.03	34.33	0.77	2.58	33.29
	at.%	44.73	39.71	0.99	1.70	12.87

**Table 2:** Effect of TiO_2_ particle size and epoxy-resin content on WCA and SA. The 50–100 nm TiO_2_/2 wt.% EP formulation was selected as the optimized coating for subsequent tests.

EP (wt.%)	20 nm WCA (°)	20 nm SA (°)	50–100 nm WCA (°)	50–100 nm SA (°)	1 μm WCA (°)	1 μm SA (°)
0	153.0	6.5	157.4	4.3	153.3	7.8
1	152.4	7.2	156.6	5.2	154.1	12.5
2	151.7	8.9	156.4	6.9	152.5	21.5
5	147.6	25.1	153.3	7.5	139.7	—
10	137.4	—	151.9	13.8	128.8	—
15	121.9	—	145.6	27.5	114.6	—

**Table 3:** Sample codes used in the epoxy-content comparison.

Sample Code	EP Content (%)	TiO_2_ Particle Size
Original bamboo	-	-
TOB	0	50–100 nm
T2EOB	2	50–100 nm
T5EOB	5	50–100 nm
T10EOB	10	50–100 nm

**Table 4:** Flexural-strength retention after 672 h xenon-lamp aging.

Sample	Before Aging σb (MPa)	After 672 h σb (MPa)	Retention (%)
Original bamboo	197.4	155.4	78.7
EP/TiO_2_/ODTMS	225.7	205.6	91.1

## Data Availability

The original contributions presented in this study are included in the article/Supplementary Materials. Further inquiries can be directed to the corresponding author.

## Supplementary Materials

The following supporting information can be downloaded at https://www.mdpi.com/article/10.3390/molecules31162773/s1, Figure S1: Representative photographs of the mechanical-durability tests for EP/TiO_2_/ODTMS-coated bamboo: (a) calibration of the 100 g applied load and abrasion distance; (b) sandpaper-abrasion test under a 100 g load; (c) overall setup of the gravel-impact test; and (d) gravel-impact process on the coated bamboo surface.
