# Supplementary material for: Mechanism-Guided Spray Deposition of Rutile TiO2/Epoxy/ODTMS Superhydrophobic Coatings for Weather-Resistant Bamboo Sand Barriers
Source: Molecules. 2026 Aug 10;31(16):2773. doi: 10.3390/molecules31162773 (PMC13515753; doi:10.3390/molecules31162773)
Supplement: Supplementary file 1 [file molecules-31-02773-s001.zip › molecules-4448057-supplementary.pdf]

Supplementary Figure S1

Mechanism-Guided Spray Deposition of Rutile TiO_2/Epoxy/ODTMS Superhydrophobic Coatings for Weather-Resistant Bamboo Sand Barriers

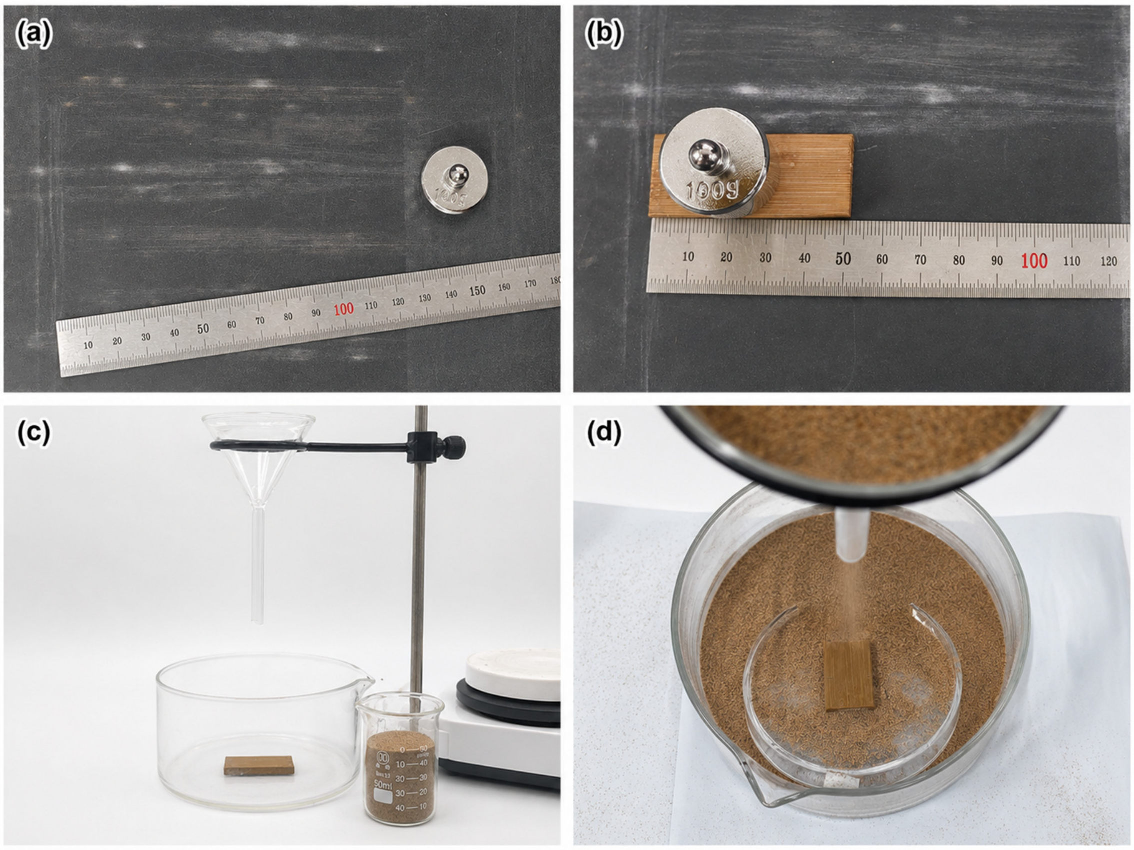

Figure S1. Representative photographs of the mechanical-durability tests for EP/TiO_2/ODTMS-coated bamboo: (a) calibration of the 100 g applied load and abrasion distance; (b) sandpaper-abrasion test under a 100 g load; (c) overall setup of the gravel-impact test; and (d) gravel-impact process on the coated bamboo surface.
